# Pannus inflammation in sacroiliitis following immune pathological injury and radiological structural damage: a study of 193 patients with spondyloarthritis

**DOI:** 10.1186/s13075-018-1594-z

**Published:** 2018-06-08

**Authors:** Dan min Wang, Ling Lin, Jian hua Peng, Yao Gong, Zhi duo Hou, Su biao Chen, Zheng yu Xiao

**Affiliations:** 1 Department of Rheumatology, the first Affiliated Hospital of Shantou University Medical College, No.57 Chang ping Road, Shantou, 515041 Guangdong Province China; 2 Department of Rheumatology of Shantou University Medical College, No.22 Xin Ling Road, Shantou, 515041 Guangdong Province China

**Keywords:** Spondyloarthritis, Sacroiliitis, Immunopathology, Pannus

## Abstract

**Background:**

The pathogenesis of sacroiliitis is unclear; therefore, we aimed to systematically study the immunopathology of sacroiliitis in patients with axial spondyloarthritis (axSpA), and explore the relationship between pannus formation, inflammation, and the structural damage caused by sacroiliitis.

**Methods:**

Fine needle aspiration biopsy of the sacroiliac joint (SIJ) was performed in 193 patients with axSpA. Clinical, laboratory, and imaging data were collected at baseline and during the follow up. Immunohistochemistry analysis was performed to detect CD34+ microvessels, CD68+ osteoclasts/macrophages, vascular endothelial growth factor (VEGF), metalloproteinase-3 (MMP-3), tumor necrosis factor-α (TNF-α), and caspase-3. Autopsy subjects were used as controls.

**Results:**

In early sacroiliitis (grade 0–1) all pathological features could be observed, with the most common being subchondral pannus formation. Among the 193 patients, 98 were followed up for 1–13 years (mean 3.6 years); 63.3% had radiological progression at the endpoint. Multiple regression analysis showed that cartilage pannus invasion (OR 2.99, *P* = 0.010) and endochondral ossification (OR 3.97, *P* = 0.049) at baseline were risk factors for radiological structural damage. Compared to SIJ controls, the subchondral microvessel density, number of CD68+ multinuclear osteoclasts, and the levels of VEGF, caspase-3, MMP-3, and TNF-α expressed at the interface of the bone and cartilage were significantly higher in patients with sacroiliitis.

**Conclusions:**

Subchondral fibrovascular tissue formation is the most important pathological feature in early sacroiliitis. The existence of cartilage pannus invasion or endochondral ossification at baseline can predict radiological structural damage during the follow up.

**Electronic supplementary material:**

The online version of this article (10.1186/s13075-018-1594-z) contains supplementary material, which is available to authorized users.

## Significance and innovations

1. This study aimed to systematically study the immunopathology of sacroiliitis in patients with axial spondyloarthritis (axSpA), and to explore the relationship between pannus formation, inflammation, and the structural damage caused by sacroiliitis. We found that subchondral pannus formation was the most significant manifestation of early sacroiliitis.
2. Subchondral pannus invasion was central to the pathological degeneration of the cartilage.
3. The existence of cartilage pannus invasion or endochondral ossification at baseline can predict radiological structural damage during the follow up.

## Background

The sacroiliac joint (SIJ) is situated where the lower spine and the pelvis connect. It is the area initially affected in axial spondyloarthritis (axSpA), which is a type of chronic inflammatory arthritis involving the SIJ. Research on the histopathology of sacroiliitis is in its infancy due to difficulties in obtaining raw materials. There are only a few studies on the immunopathology of SIJ in patients with axSpA , particularly in patients with early disease, which show inconsistent findings [1–6].

At the Department of Rheumatology (Shantou University Medical College, Shantou, China), we have studied approximately 200 SIJ biopsies guided by computed tomography (CT), since 2000. New standards for diagnosing pathologic changes within the SIJ have advanced over the last 10 years. These new diagnostic criteria include the radiology sacroiliac arthritis grade ≤ 1, along with the presence of one or more of the following three inflammatory processes: (i) subchondral bone marrow edema and fibrous connective tissue proliferation with mononuclear cell infiltration and pannus formation; (ii) subchondral bone plate destruction with pannus invasion; or (iii) cartilage pannus invasion [4].

In this study, we assessed the relationship between pannus formation, inflammation, and the structural damage to the SIJ by analyzing the pathologic changes in different grades of sacroiliitis. Furthermore, we explored the related pathogenic mechanism of sacroiliitis to enable identification of markers for the early diagnosis and treatment of axSpA.

### Research Methods

#### Subjects

We obtained a biopsy of the SIJ from patients diagnosed with axSpA. Non-SpA control biopsies were obtained at autopsy. One hundred and ninety three patients with axSpA underwent a fine needle aspiration biopsy of the SIJ at the Department of Rheumatology (First Affiliated Hospital, Shantou University Medical College), from 2000 to 2013. All diagnoses were made in accordance with the axSpA classification criteria [7, 8], which were proposed by the Assessment of Spondyloarthritis International Society (ASAS) in 2009.

Twelve SIJ autopsy specimens were obtained from autopsies in patients between 15 and 44 years of age (10 male and 2 female) who exhibited no history of SpA. The causes of death included drug allergy, violent lesion, pesticide poisoning, severe acute pancreatitis, pulmonary embolism, cerebral hemorrhage, inflammatory response syndrome, cardiomyopathy, myocarditis, and pulmonary tuberculosis.

## Methods

### Clinical data collection

General clinical data from patients with axSpA who underwent SIJ fine needle aspiration biopsy were collected, including age, gender, course of the disease, initial site, and symptoms. Laboratory tests included measurement of human leukocyte antigen B27 (HLA B27), erythrocyte sedimentation rate (ESR), and C-reactive protein (CRP) levels. Radiographs of the pelvis and lumbar spine, and CT/magnetic resonance imaging (MRI) data on the SIJ were also obtained.

### Diagnostic criteria

The diagnosis of axSpA was made according to the axSpA classification criteria established by the ASAS, as revised in the New York criteria [9]. Subjects with axSpA who did not exhibit radiological sacroiliitis and did not meet the New York criteria were defined as having non-radiographic axSpA (nr-axSpA) [10].

### MRI edema scoring

The degree of bone marrow edema in the SIJ was graded using the Spondyloarthritis Research Consortium of Canada criteria [11]. Oblique coronal plane scanning was adopted by selecting six consecutive synovial areas (sacrum and sacral foramen of the lateral sacrum, not including synovial and ligaments). Each joint was divided into four quadrants that constituted the upper and lower part of the ilium and sacrum at all levels. Any quadrant with bone marrow edema received a score of 1, while 0 represented the absence of edema. If the signals of edema were similar to those of blood vessels, then one point was added to this score. If the range of edema below the bone lamella was greater than 1 cm, then an additional point was added to this score. The highest possible score for a quadrant was 12, with the total possible score being 72.

### Fine needle aspiration biopsy

Patients in the prone position underwent a biopsy of the SIJ using an FRANSEEN lung biopsy needle (Cook Company, DFBN - 15 or 16–18-15), guided by CT. The intra-articular tissues were extracted by negative pressure [4, 12]*.*

### Autopsy specimens

The pelvis was exposed by lateral cutting of the abdomen. The psoas major muscle was cut and stripped toward its site of attachment and as far laterally as possible, thereby exposing the upper part of the SIJ. A saw was used to mutilate both ends of the SIJ away from the articular surface by approximately 2 cm and then the anatomical ends were cut and immediately immersed in 4% neutral formalin.

### Tissue processing

All specimens were fixed with 10% formalin solution, decalcified with 10% EDTA solution, dehydrated by an automatic tissue hydroextractor (type: CJ - 14 d2, Dingyuan Hubei Co., Ltd), embedded in paraffin, and sectioned into 4-μm-thick slices.

### Immunohistochemistry analysis

Kit I included antibodies against CD34, CD68, vascular endothelial growth factor (VEGF), caspase-3 (rat monoclonal antibody, Zho Zhongshan Gene Co., Ltd., Beijing), type I collagen (rabbit polyclonal antibody. Zhongshan Gene Co., Ltd., Beijing, China), metalloproteinase-3 (MMP-3; rabbit polyclonal antibody; Abcam Co., Ltd., USA), and tumor necrosis factor-α (TNF-α; rabbit polyclonal antibody; Boster Co., Ltd., Wuhan, China). Immunohistochemistry kit II included a two-step mouse and rabbit ultra-sensitive PV immunohistochemistry ELISA kit with a two-step (polymer auxiliary agent + IgG polymer labeled with horseradish enzyme; Zhongshan Gene Co., Ltd., Beijing, China) SAP immunohistochemistry detection kit (blocked with normal sheep serum, goat anti-mouse/rabbit IgG labeled with biotin and chain mildew-avidin labeled with alkaline phosphatase; Zhongshan Gene Co., Ltd., Beijing, China). Staining reagents included ready-to-use hematoxylin dye solution (Huntz Enterprises Inc., Shanghai, China), eosin (Solarbio Inc., Shanghai, China), and safranin and fast green stain solution kits (IHC World Co., Ltd., USA). All sections were stained with hematoxylin-eosin and safranin O-fast green. Some of sections were stained using the immunohistochemistry reagents described above. Slides were incubated with primary antibody overnight at 4 °C, and with secondary antibody for 15 min at 37 °C. Slides were then visualized with 3,3-diaminobenzidine (DAB) or AP-Red.

### Microscopic examination

The various pathologic changes in the biopsy specimens were observed using microscopy. These changes were compared with healthy autopsy specimens.

### Definition of pathologic changes in sacroiliitis

Pathologic changes were defined as follows:

1. Cartilage degeneration: chondrocyte hyperplasia or hypertrophy, or focal distribution; cartilage matrix depletion (stained with hematoxylin-eosin and safranin O-fast green), or fibrosis and mucoid degeneration.
2. Endochondral ossification: bone deposits on remnants of the cartilage matrix.
3. Pannus formation: highly vascular granulation tissue formed from the inflamed synovium or subchondral bone marrow.
4. Subchondral bone disruption: pannus invasion and destruction of the subchondral bone.
5. Osteoclast activation: formation of at least five CD68+ multinuclear osteoclasts in areas of bone resorption at the subchondral bone endplate or at the bone-cartilage interface, which are expressed as the total number per 10 high-power fields (hpf).
6. Sequestrum: a fragment of bone that has become necrotic and has separated from the normal bone structure.
7. Pathologic new bone formation: at the inflamed bone-cartilage interface, accompanied with granulation tissue, new bone tissue forms and is surrounded by osteoblastic layers.
8. Marrow inflammatory cell infiltration: mononuclear cells (CD3+ T cells or CD68+ macrophages) aggregated in the subchondral bone marrow, defined as ≥ 50 per 10 hpf.
9. Synovitis: inflammation of the synovium with hyperplasia of the synovial lining cells and hyperplasia of the loose connective tissues and interstitial edema, which can be accompanied by inflammatory cell infiltration.
10. Enthesitis: iInflammation of the enthesis, with highly vascular dense connective tissue (ligament) and inflammatory cell infiltration.

The density of the micro-vessels (marked with CD34) and the numbers of CD68+ osteoclasts/macrophages at the conjunctional zone of the cartilage and the bone plate were calculated from five continuous visual fields; the number of microvessels and osteoclasts/macrophages were counted and averaged. If the microvessel density was equal to or greater than 10 per 40 hpf, it was defined as an increase in angiogenesis.

VEGF, MMP3, TNF-α and caspase-3 expression was semiquantitatively evaluated from five visual fields (10 × 20 magnification), collected continuously at the site of the cartilage and the bone plate, and at the sites of bone marrow beneath the bone plate. We studied the biopsy specimens and evaluated the ratio of cells with positive expression in each visual field. A ratio ≤ 5% was scored as 1 point; 6–25% was scored as 2 points; 26–50% was scored as 3 points; 51–75% was scored as 4 points; and > 75% was scored as 5 points. We also employed a quantitative method of assessing expression using Image-Pro Plus to calculate the area of positive expression in each visual field (10 × 20 magnification under the same condition of exposure). The average score for multiple visual fields was then determined.

### Statistical analysis

SPSS 17.0 software was used for statistical analysis. The chi-square (χ2) test was applied to analyze the enumeration data. Measurement data are expressed as mean ± standard deviation and the normality of the measurement data was first tested; the *t* test was applied to normally distributed data and the rank sum test was used for non-normally distributed data. Binary multivariate regression analyses were applied to analyze the factors that influence the progression of SIJ imaging in different pathologic manifestations of cartilage and bone plate. The Mann-Whitney U test was used to analyze the immunohistochemical results. Statistical differences were assumed to be significant when the *p* value was < 0.05.

## Results

### General clinical data

Included in the study were 193 patients with axSpA, with a male-to-female ratio of 2.3:1, an average age of 23.5 ± 8.9 years, and an average disease course of 4.0 ± 3.8 years. The positive rate of HLA-B27 was 63.4% in the patients with axSpA. Additional file 1: Table S1 shows the general clinical data in more detail.

### Pathology of sacroiliitis

**Fig. 1:**
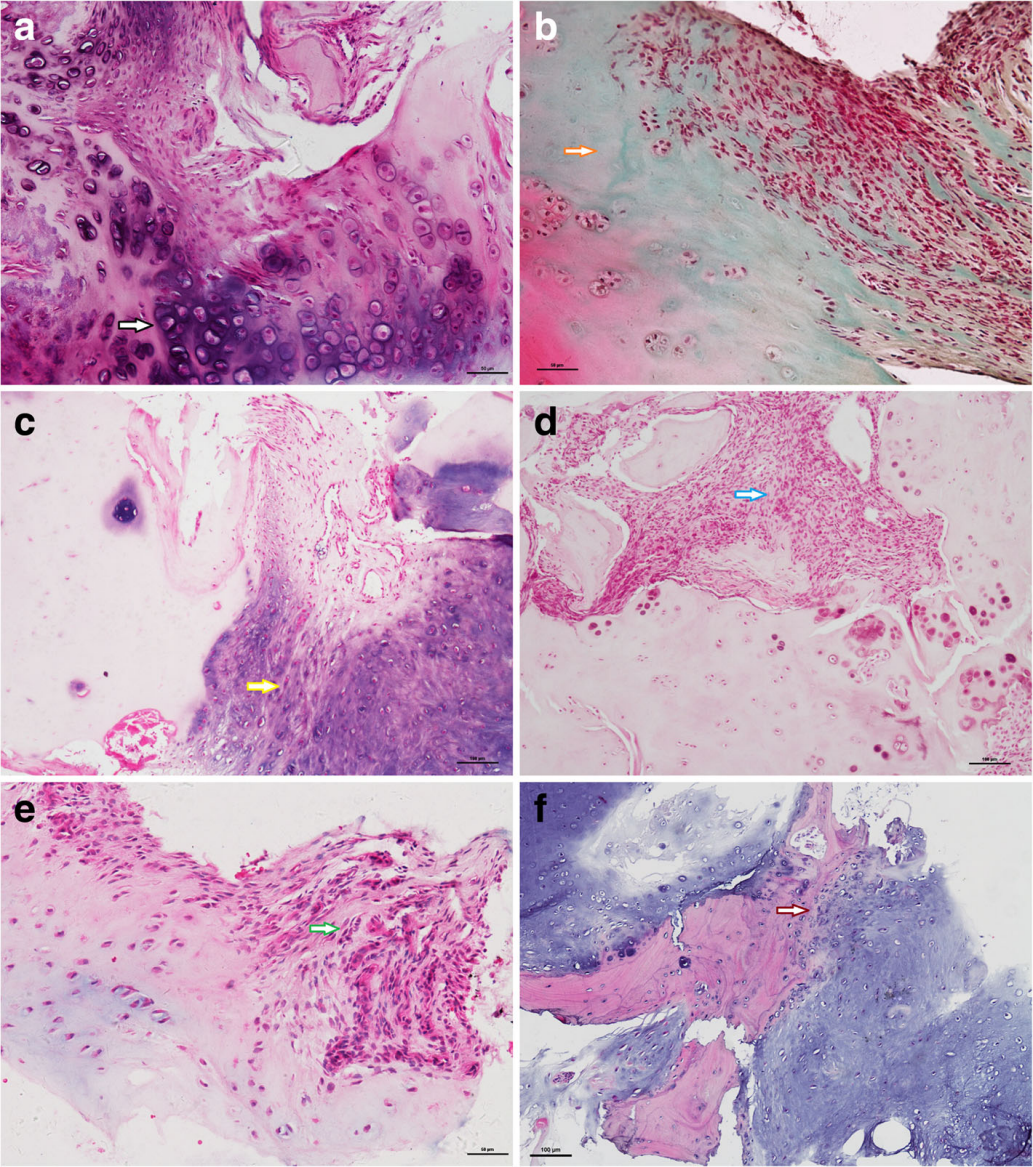
Pathologic features of the cartilage in sacroiliitis (hematoxylin-eosin staining **(a**, **c**-**f),** safranin O-fast green staining (**b**)). **a** Subchondral pannus invasion into the cartilage, accompanied with chondrocyte hypertrophy degeneration (arrow with black outline). **b** Pannus invasion resulted in cartilage matrix depletion (arrow with orange outline). **c** Neovascular formation and fibrotic cartilage (arrow with yellow outline). **d** Granulation tissue repaired the destroyed cartilage (arrow with blue outline). **e** Synovium pannus that has invaded into the cartilage surface (arrow with green outline). **f** Endochondral ossification (arrow with red outline)

**Fig. 2:**
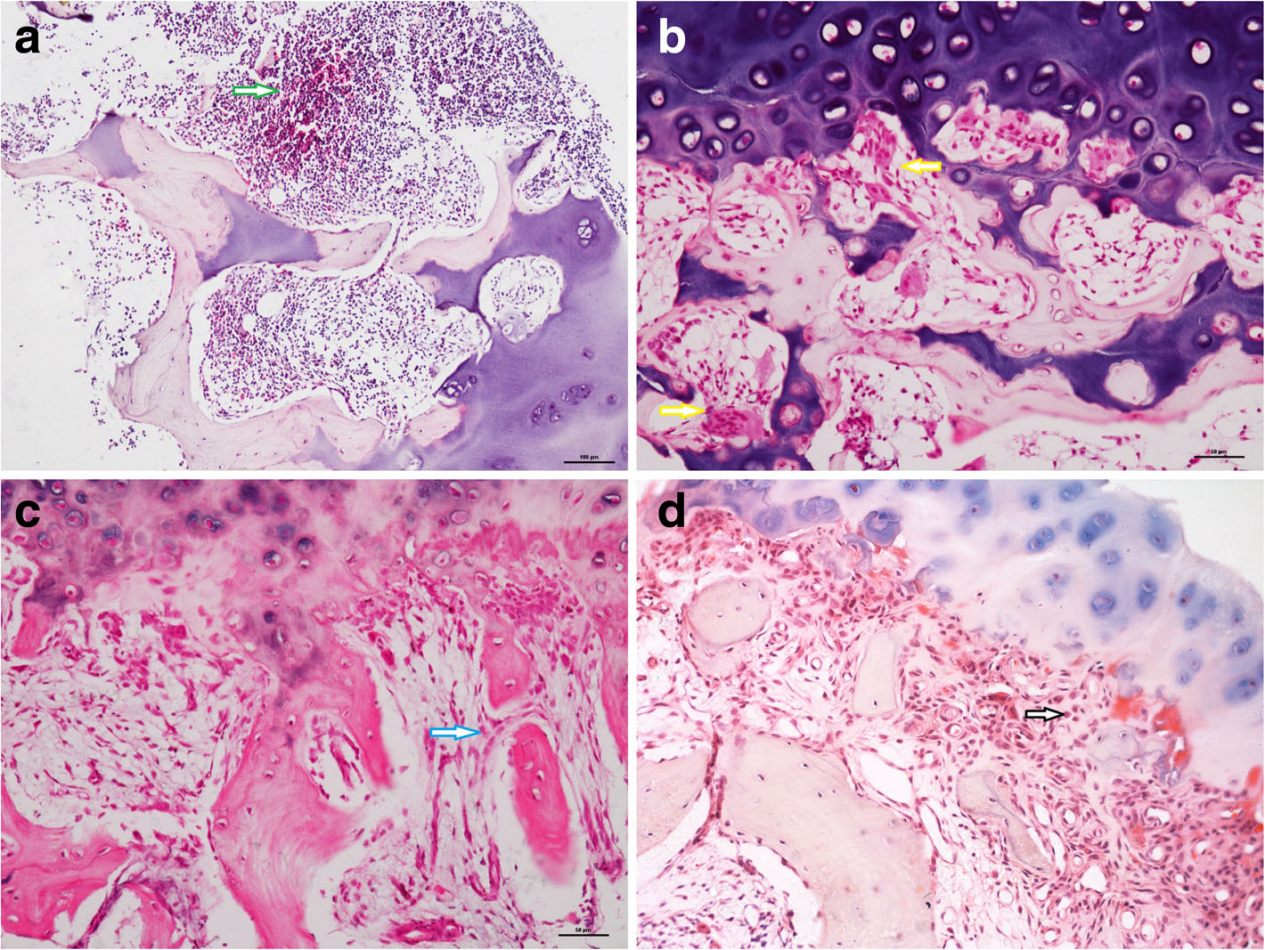
Pathologic features of the subchondral bone (hematoxylin-eosin staining). **a** Marrow inflammatory cell infiltration (arrow with green outline). **b** Subchondral osteoclast activation (arrow with yellow outline). **c** Subchondral fibrovascular tissue formation with bone plate disrupted (arrow with blue outline). **d** Abundant fibrovascular tissue formation and invasion into the cartilage (arrow with black outline)

Patients with axSpA underwent CT-guided, fine-needle aspiration biopsy of the SIJ. The acquisition rates of cartilage, bone plate, bone marrow, synovial membrane, and enthesitis were 83.9%, 62.0%, 40.0%, 8.8%, and 14.0%, respectively. As shown in Figs 1 and 2, the pathologic changes during SIJ inflammation included chondrocyte and cartilage matrix degeneration, cartilage pannus invasion, endochondral ossification, subchondral pannus formation, subchondral bone disruption, sequestrum, osteoclast activation, pathologic new bone formation, marrow inflammatory cell infiltration, synovitis, and enthesitis. The incidence of pathologic changes was significantly higher than in the control autopsy specimens. Additional file 2: Table S2 shows this in more detail.

**Fig. 3:**
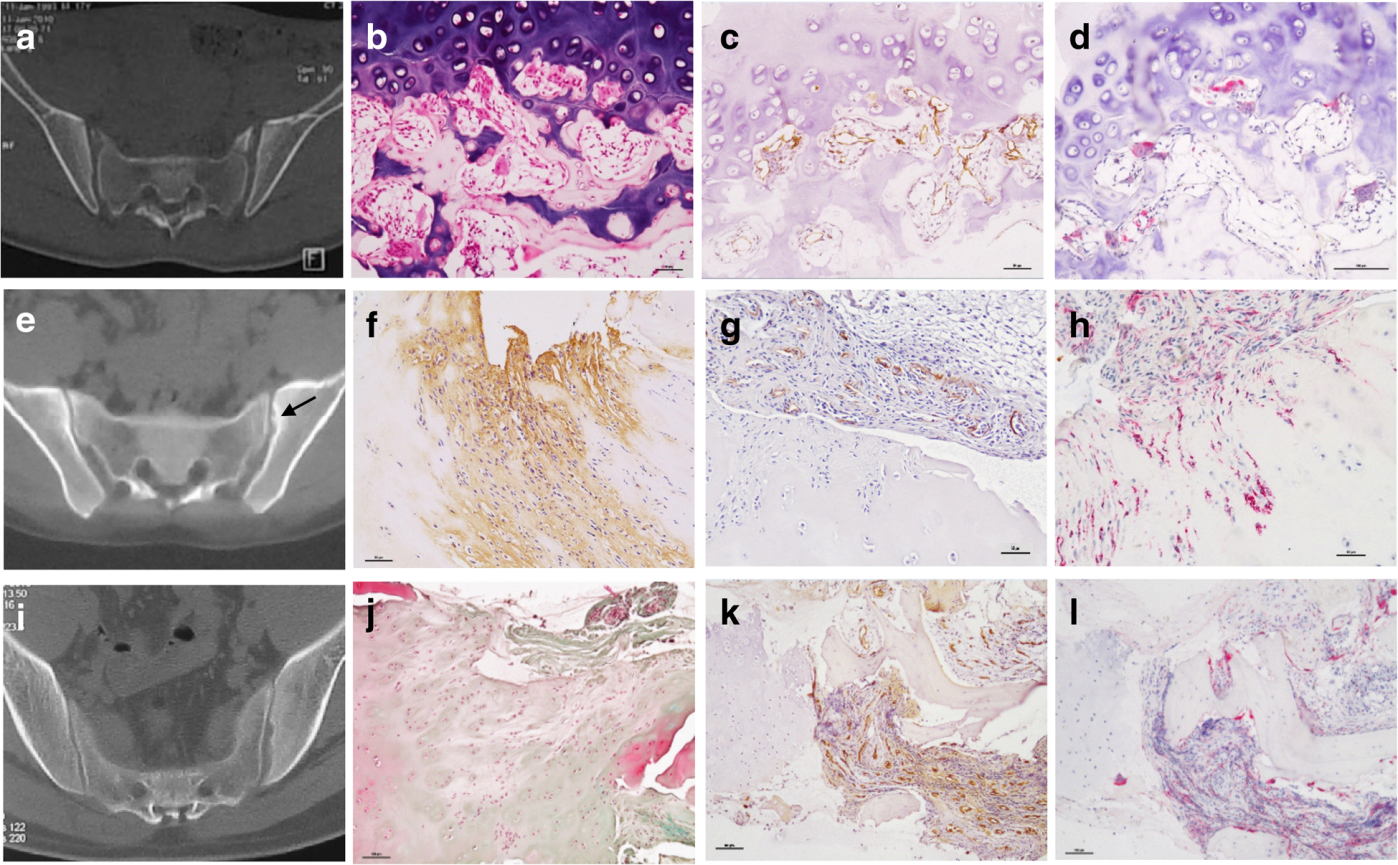
Immune pathologic changes in different grades of sacroiliitis. **a**-**d** In grades 0–1 sacroiliitis (**a**), inflammatory cells have infiltrated and subchondral fibrovascular tissue has formed without definite structural damage (**b**); immunohistochemistry staining showed that CD34+ microvessel (**c**) and CD68+ osteoclasts (**d**) increased in subchondral bone. **e-h** In grade 2 sacroiliitis, pannus invasion resulted in cartilage fibrosis degeneration (**f**, type I collagen immunohistochemistry staining); cavity side pannus formed and invaded into cartilage (**g**, CD34 immunohistochemistry staining), accompanied by endochondral ossification; abundant CD68+ macrophages expressed in the cartilage surface where pannus invaded (**h**). **i-l** In grade 3 sacroiliitis, pannus invasion resulted in cartilage degeneration (chondrocytes hyperplasia and cartilage matrix depletion) (**j**), abundant fibrovascular tissue formed (**k**, CD34+) and destroyed the subchondral bone, abundant CD68+ osteoclasts were expressed in areas of bone resorption (**l**)

The inflammatory cell infiltration decreased in a manner that was commensurate with an increase in radiographic sacroiliitis, structural cartilaginous damage, and bone plate damage (Table 1). In specimens with a level of radiographic sacroiliitis below II, the pathologic manifestations of arthritis varied, but all exhibited structural damage. For example, the ratio of pannus and granulation tissue in subchondral bone was elevated and was accompanied by inflammatory cell infiltration. The ratio of cavity side pannus invasion during the early stages of sacroiliitis was less than 5%, while the rates of synovitis and enthesitis in early sacroiliitis were 29.6% and 16.1%, respectively. The immune pathologic changes in different grades of sacroiliitis are summarized in Fig. 3.

**Table 1:** Pathological changes in different grades of sacroiliitis

	Sacroiliitis < 2 (*N* = 271)	Sacroiliitis ≥2 (*N* = 115)	*P*
Chondrocyte degeneration, *n*/n* (%)	52/234 (22.2)	55/90 (61.1)	*0.000*
Cartilage matrix degeneration, *n*/n* (%)	103/234 (44.0)	70/90 (77.8)	*0.000*
Cartilage pannus invasion, *n*/*n* * (%)	61/234 (26.1)	52/90 (57.8)	*0.000*
Cavity side pannus invasion, *n*/*n* * (%)	7/234 (3.0)	12/90 (13.3)	*0.001*
Marrow side pannus invasion, *n*/*n* * (%)	57/234 (24.4)	51/90 (56.7)	*0.000*
Endochondral ossification, *n*/*n* * (%)	15/234 (6.4)	25/90 (27.8)	*0.000*
Subchondral pannus formation, *n*/*n* * (%)	134/174 (77.0)	70/75 (93.3)	*0.001*
Subchondral bone disruption, *n*/*n* * (%)	128/174 (73.6)	69/75 (92.0)	*0.001*
Osteoclast activation, *n*/*n* * (%)	81/174(46.6)	37/75 (49.3)	0.065
Pathologic new bone formation, *n*/*n* * (%)	47/174 (27.0)	29/75 (38.7)	0.155
Sequestrum, *n*/*n* * (%)	7/174 (4.0)	13/75 (17.3)	*0.000*
Marrow inflammatory cell infiltration, *n*/*n* * (%)	57/108 (52.8)	19/54 (35.2)	*0.017*
Synovitis, *n*/*n* * (%)	8/27 (29.6)	4/6 (66.7)	0.173
Enthesitis, *n*/*n* * (%)	5/31 (16.1)	11/23 (47.8)	*0.019*

*SIJ* sacroiliac joint, n* number of specimens that contain the corresponding tissues

### Relationship between SIJ radiological structural damage progression during follow up and the manifested pathologic appearances at baseline

Among the 193 patients with axSpA, 98 (29 with ankylosing spondylitis (AS) and 69 nr-axSpA) had a follow-up period of 1–13 years, with the average follow-up time being 3.6 ± 2.7 years. Except in three patients who did not follow regular treatment, the other 95 patients followed their doctor’s advice to use traditional anti-rheumatic drugs. There were 62 patients (approximately 63.3%) with progression of structural damage upon radiographic assessment, including two patients who had received irregular treatment. Among the patients with AS, 86.2% exhibited radiological progression, compared to 53.6% of patients with nr-axSpA, of whom 19 (27.5%) eventually developed AS. The proportion of various structural pathologic changes in cartilage and the bone plates in the radiographic progression group was higher than that in the non-radiographic progression group. The proportion of pathologic fibrosis of cartilage (14.7% vs. 2.7%), the invasion of subchondral pannus (49.5% vs. 17.8%), endochondral ossification (22.1% vs. 4.1%), and the formation of pannus/granulation in the bone plate (89.2% vs. 74.5%) was statistically significant between the two groups.

Significant differences in pathologic manifestation and follow up time between the two groups, as indicated by single factor regression analysis (*P* < 0.1), were further analyzed using logistic regression analysis. The results demonstrated that a long follow-up time (OR 1.17, *P* = 0.038), medulla pannus invasion of the cartilage (OR 2.99, *P* = 0.010), and endochondral ossification (OR 3.97, *P* = 0.049) were risk factors for SIJ inflammation.

### Pannus invasion and related inflammation

**Fig. 4:**
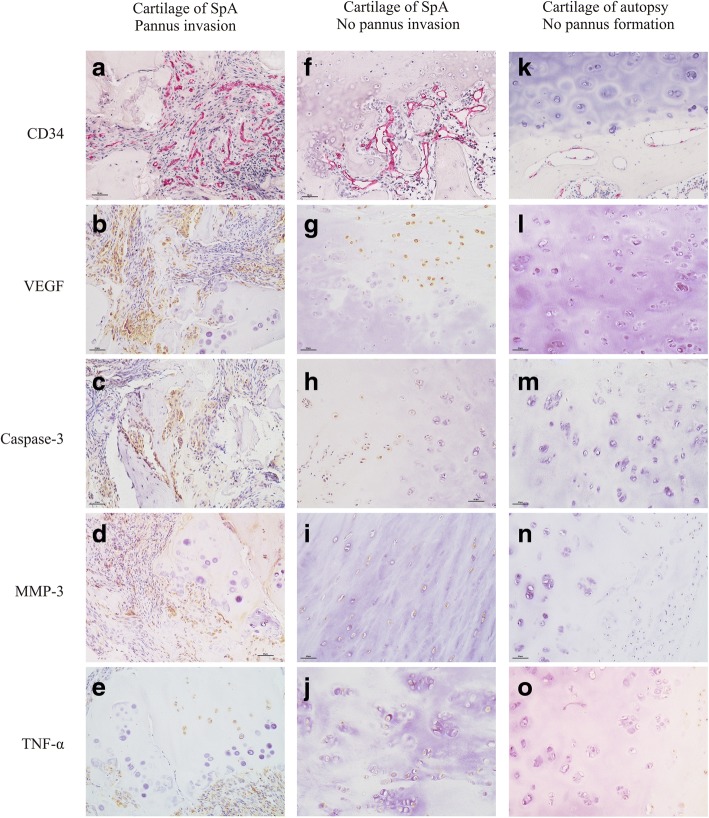
Influence of the invasion of pannus in the cartilage area on cartilaginous structures and related inflammation expression (10 × 20 magnification). **a**-**e** In sacroiliitis, abundant fibrovascular tissue formed and invaded into cartilage, chondrocytes and matrix degenerated (**a**), accompanied by high levels of vascular endothelial growth factor (VEGF) (**b**), caspase-3 (**c**), matrix metalloproteinase-3 MMP-3 (**d**) and TNF-α (**e**) expressed in the cartilage. **f**-**j** In sacroiliitis, fibrovascular tissue formed in the subchondral area without invading into cartilage (**f**), accompanied by significantly lower levels of VEGF (**g**), caspase-3 (**h**), MMP-3 (**i**) and TNF-α (**j**) expressed in the cartilage. **k**-**o** In autopsy controls, there was no fibrovascular tissue formation and only partial superficial chondrocytes expressed low levels of VEGF (**l**), caspase-3 (**m**) and TNF-α (**o**). **a**, **f**, **k**, AP-Red staining; **b**-**e**, **g**-**j**, **l**-**o**, 3,3-diaminobenzidine staining. SpA, spondyloarthritis

To study the influence of pannus invasion into the cartilage and the related inflammatory response, the groups were divided based upon the degree of vascular invasion into the cartilage area, into a pannus invasion group and a non-pannus invasion group. As shown in Table 2 and Fig. 4, the proportion of patients exhibiting pannus inside the cartilage that also had chondrocyte degeneration (79.6%), cartilage matrix degeneration (88.5%), and endochondral ossification (38.5%) was significantly higher than in the non-pannus invasion group (20.8%, 37.5%, and 20.8%, respectively). The pannus invasion group exhibited increased expression of VEGF, caspase-3, MMP-3 and TNF-α in the cartilage. Therefore, with the exception of caspase-3, all inflammatory factors were significantly elevated with vascular invasion. The increased expression of MMP-3 was accompanied by distinct attrition in the cartilage matrix.

**Table 2:** Influence of the invasion of pannus in the cartilage area on cartilaginous structures and related inflammation expression

	Pannus invasion (*N* = 26)	No pannus invasion (*N* = 24)	*P*
Cartilage			
Chondrocyte degeneration, *n* (%)	20 (76.9)	5 (20.8)	*0.000*
Cartilage matrix degeneration, *n* (%)	23 (88.5)	9 (37.5)	*0.000*
Endochondral ossification, *n* (%)	10 (38.5)	5 (20.8)	0.174
VEGF			
Scores	2.0 ± 1.3	1.1 ± 1.2	*0.021*
Area of positive expression (μm^2^)	657.4 ± 984.9	199.3 ± 418.8	*0.022*
Caspase-3			
Scores	2.3 ± 0.9	1.8 ± 1.7	0.419
Area of positive expression (μm^2^)	620.0 ± 652.5	207.7 ± 285.2	0.185
MMP-3			
Scores	2.0 ± 1.1	0.7 ± 0.8	*0.010*
Area of positive expression (μm^2^)	378.4 ± 406.0	50.3 ± 83.3	*0.008*
TNF-α			
Scores	3.2 ± 1.2	1.6 ± 1.0	*0.012*
Area of positive expression (μm^2^)	846.9 ± 739.2	181.1 ± 172.8	*0.025*
Bone-cartilage interface (*n*/40 hpf)			
CD34+ microvessels	12.7 ± 3.6	7.4 ± 2.0	*0.000*
CD68+ macrophages	10.1 ± 2.9	8.7 ± 3.5	0.277
CD68+ multinuclear osteoclasts	2.3 ± 0.9	1.9 ± 0.9	0.228

*VEGF* vascular endothelial growth factor, *MMP* matrix metalloproteinase, *TNF* tumor necrosis factor, *hpf* high power fields

At the bone-cartilage interface, the density of the CD34+ microvessels and the number of CD68+ macrophages and CD68+ multinuclear osteoclasts were higher in the pannus invasion group than those in the non-pannus invasion group (Table 2).

## Discussion

In the clinic, we observed dramatically different outcomes among various patients with nr-axSpA. Most of these patients eventually progress into AS during follow up [4], whilst there are large numbers of patients who do not exhibit clear manifestation of radiological structural changes. By studying the relationship between pannus formation, inflammation, and structural damage to the SIJ during follow up, our results indicate that the formation of a medullary lateral pannus in tissues with pathologic change, resulting in pannus invasion of the cartilage and endochondral ossification, was a risk factor for the progression of radiological sacroiliitis.

The pathogenic mechanism of AS has not yet been fully elucidated and there are many hypotheses on the starting position of inflammation in AS. Early theories postulated that the pathologic manifestation of arthritis in AS is similar to that in rheumatoid arthritis (RA), wherein the synovium is the most affected area [13, 14]. Conversely, others have argued that the pathologic mechanism of AS is different from that of RA, wherein the foremost affected region is the entheses [15–18]. However, the relevant evidence for this argument mainly came from evidence in the spine and peripheral joints, while the SIJ region was less involved.

Presently, more evidence [3, 4, 19–27] supports the observation that the foremost part of AS immunological pathologic change includes subchondral bone inflammation in the areas where bone and cartilage appose one another. Our research found that, in the early stage of sacroiliitis, pannus formation in subchondral tissue may be an early pathological hallmark. Subsequently, inflammatory cell infiltration, progression of the pannus, damage of the bone plate, and invasion into the cartilage occurs. Among specimens from patients with sacroiliitis at levels 0–1, the incidence of cartilage damage triggered by the intrusion of a cavity side pannus was less than 5%, which suggests that SIJ inflammation originated from the subchondral bone and then progressed upwards. Ultimately, the inflammation damaged the surface of the SIJ by narrowing or integrating into the articular cavity. The occurrence rates of synovitis and enthesitis were not high in patients with level 0–1 sacroiliitis. It is possible that there is a somewhat causal relationship between the infiltration of inflammation and pannus formation. The inflammation may trigger the formation of angiogenesis; however, increased microvessel density may provide a channel for inflammatory cell invasion and inflammatory mediator expression, which in turn aggravates the regional damage of inflammation.

This study found that the microvessel density in the cartilage-bone interface was significantly increased in patients with sacroiliitis. Pannus invasion resulted in cartilage fibrosis degeneration and endochondral ossification, and the regional expression of VEGF, caspase-3, MMP3, and TNF-α were significantly increased. The immunochemistry analysis of the articulation of the os coxae in patients with AS, RA and osteoarthritis (OA), conducted by Appel et al. [27], demonstrated that microvessel density in the AS cartilage-bone interface was significantly higher than in patients with RA and OA (13.4/hpf vs. 7.5/hpf vs. 4.7/hpf). Various studies in SpA peripheral arthritis have also indicated that angiogenesis in SpA peripheral articular synovium dramatically increases to a level higher than in the articular synovium in RA [28–30]. This increase in angiogenesis has been shown to decrease significantly after TNF-α antagonist treatment, demonstrating the important role of angiogenesis in AS. In inflamed joints, the increased density of capillaries below the cartilage or the progression of angiogenesis into the cartilage, may serve as a signaling pathway for the invasion of inflammatory cells and medium, which could further trigger damage to the cartilage. Previous studies have demonstrated that neo-angiogenesis in the cartilage growth plate plays a fundamental role in endochondral ossification [31–33].

## Conclusions

In conclusion, subchondral pannus formation was the most significant manifestation of early sacroiliitis. Furthermore, its invasion was central to the pathologic degeneration of the cartilage. Patients with side marrow pannus and granulation invasion into the cartilage or endochondral ossification are more likely to suffer from radiological structural damage in sacroiliitis.

## Additional files

Additional file 1: **Table S1.** General clinical data on the 193 patients with axSpA. Among the patients with axSpA , 49 had ankylosing spondylitis (AS) and the other 144 had nr-axSpA. Compared to patients with nr-axSpA, patients with AS were older, had a longer disease course, and experienced a higher level of night pain, and the morning stiffness ratio, HLA-B27 positive rate, and SIJ-MRI positive rate of edema were significantly higher. (DOCX 15 kb)

Additional file 2: **Table S2.** Comparison of pathologic changes between the axSpA group and autopsy controls. The incidence of pathologic changes including chondrocyte and cartilage matrix degeneration, cartilage pannus invasion, endochondral ossification, subchondral pannus formation, subchondral bone disruption, sequestrum, osteoclast activation, pathologic new bone formation, marrow inflammatory cell infiltration, synovitis, and enthesitis were significantly higher in the axSpA group than in the control autopsy specimens. (DOCX 14 kb)

## Abbreviations

AS: Ankylosing spondylitis
ASAS: Assessment of Spondyloarthritis International Society
axSpA: Axial spondyloarthritis
CRP: C-reactive protein
CT: Computed tomography
DAB: 3,3-Diaminobenzidine
ELISA: Enzyme-linked immunosorbent assay
ESR: Erythrocyte sedimentation rate
HLA: Human leukocyte antigen
HP: High power microscopic
MMP-3: Matrix metalloproteinase-3
MRI: Magnetic resonance imaging
Nr-axSpA: Non-radiographic axial spondyloarthritis
RA: Rheumatoid arthritis
SIJ: Sacroiliac joint
TNF-α: Tumor necrosis factor-α
VEGF: Vascular endothelial growth factor
