# Supplementary material for: Pannus inflammation in sacroiliitis following immune pathological injury and radiological structural damage: a study of 193 patients with spondyloarthritis
Source: Arthritis Res Ther. 2018 Jun 8;20:120. doi: 10.1186/s13075-018-1594-z (PMC5994024; doi:10.1186/s13075-018-1594-z)
Supplement: Supplementary file 1 — Table S1. General clinical data on the 193 patients with axSpA. Among the patients with axSpA , 49 had ankylosing spondylitis (AS) and the other 144 had nr-axSpA. Compared to patients with nr-axSpA, patients with AS were older, had a longer disease course, and experienced a higher level of night pain, and the morning stiffness ratio, HLA-B27 positive rate, and SIJ-MRI positive rate of edema were significantly higher. (DOCX 15 kb) [file 13075_2018_1594_MOESM1_ESM.docx]

**Supplementary table 1. General clinical data of the 193 cases of axSpA**

|  | axSpA  （N=193） | AS  (N=49) | nr-axSpA (N=144) | *P* |
| --- | --- | --- | --- | --- |
| Male n (%) | 134 (69.4) | 39 (79.6) | 95 (66.0) | 0.074 |
| Age (years) | 23.5±8.9 | 25.9±9.4 | 22.7±8.6 | **0.023** |
| Course of disease (years) | 4.0±3.8 | 5.4±3.6 | 3.5±3.7 | **0.000** |
| Night pain n (%) | 123 (63.7) | 37 (75.5) | 86 (59.7) | **0.047** |
| Morning stiffness n (%) | 112 (58.0) | 35 (71.4) | 77 (53.5) | **0.028** |
| HLA-B27(+) n/N1 (%) | 116/183 (63.4) | 35/43 (81.4) | 81/140 (57.9) | **0.000** |
| ESR (mm/1h) | 31.9±30.4 | 33.3±26.9 | 31.3±31.7 | 0.207 |
| CRP (mg/L) | 19.0±24.9 | 16.6±20.0 | 19.7±26.4 | 0.358 |
| SIJ-MRI edema (+) n/N2 (%) | 69/134 (51.5) | 24/27 (88.9) | 45/107 (42.1) | **0.000** |

axSpA: axial spondyloarthritis; AS: ankylosing spondylitis; nr-axSpA: nonradiographic axial spondyloarthritis; HLA: human leukocyte antigen; ESR: erythrocyte sedimentation rate; CRP: C-reactive protein; SIJ: sacroiliac joint; MRI: magnetic resonance imaging; N1: the number of patients who take HLA-B27 examination; N2: the number of patients who take SIJ MRI examination. *P*: comparison between AS group and nr-axSpA group.
