# Supplementary material for: Pannus inflammation in sacroiliitis following immune pathological injury and radiological structural damage: a study of 193 patients with spondyloarthritis
Source: Arthritis Res Ther. 2018 Jun 8;20:120. doi: 10.1186/s13075-018-1594-z (PMC5994024; doi:10.1186/s13075-018-1594-z)
Supplement: Supplementary file 2 — Table S2. Comparison of pathologic changes between the axSpA group and autopsy controls. The incidence of pathologic changes including chondrocyte and cartilage matrix degeneration, cartilage pannus invasion, endochondral ossification, subchondral pannus formation, subchondral bone disruption, sequestrum, osteoclast activation, pathologic new bone formation, marrow inflammatory cell infiltration, synovitis, and enthesitis were significantly higher in the axSpA group than in the control autopsy specimens. (DOCX 14 kb) [file 13075_2018_1594_MOESM2_ESM.docx]

**Supplementary table 2. Comparison of pathological changes between axSpA group and autopsy controls**

|  | axSpA  （N=193） | autopsy controls  （N=12） |
| --- | --- | --- |
| Chondrocyte degeneration n/n* (%) | 107/324 (33.3) | 2/13 (15.4) |
| Cartilage matrix degeneration n/n* (%) | 173/324 (53.4) | 0 |
| Cartilage pannus invasion n/n* (%) | 113/324 (34.9) | 0 |
| Endochondral ossification n/n* (%) | 40/324 (12.3) | 1/13 (7.7) |
| Subchondral pannus formation n/n* (%) | 204/249 (81.9) | 0 |
| Subchondral bone disruption n/n* (%) | 197/249 (79.1) | 0 |
| Osteoclast activation n/n* (%) | 118/249 (47.4) | 3/13 (23.1) |
| Sequestrum n /n* (%) | 20/249 (8.0) | 0 |
| Marrow inflammatory cell infiltration n/n* (%) | 76/162 (46.9) | 0 |
| Synovitis n /n* (%) | 12/34 (35.3) | 0 |
| Enthesitis n /n* (%) | 16/54 (29.6) | 0 |

axSpA: axial spondyloarthritis

n*: the number of specimens that contain the corresponding tissues.
